# 

**Published:** 1894-03

**Authors:** 


					CONtfE^TS. I?"
?fASif, I.
J
Intussusception.
By Arthur W.
Prichard, M.R.C.S. Eng.
Appendicitis.
By James Swain, M.D.,
M.S. Lond., F.R.C.S. Eng.
Bo-called " Duct Cancer" of the
Breast, with the Account of a
Case of Large Recurrent Duct
Papilloma.
By Charles A.
Morton, F.R.C.S. Eng.
23
Proge&siibf the Medical Sciences :
Meilicliltf.
By J. Michell Clarke,
M.D.
30
Surgery.
By W. M. Barclay
Otology.
By W. H. Harsant
43
Reviews of Books
45
Notes on Preparations for the Sick
Meetings of Societies
The Library of the Bristol' Medico-
Chirurgical Society   73
Scraps
79

				

